# ^18^F-FACBC PET/MRI in the evaluation of human brain metastases: a case report

**DOI:** 10.1186/s41824-021-00101-6

**Published:** 2021-04-13

**Authors:** Knut Johannessen, Erik Magnus Berntsen, Håkon Johansen, Tora S. Solheim, Anna Karlberg, Live Eikenes

**Affiliations:** 1 Department of Circulation and Medical Imaging, Faculty of Medicine and Health Sciences, Norwegian University of Science and Technology, NTNU, Postboks 8905, 7491 Trondheim, Norway; 2 Department of Radiology and Nuclear Medicine, St. Olavs Hospital, Trondheim University Hospital, Trondheim, Norway; 3 Cancer Clinic, St. Olavs Hospital, Trondheim University Hospital, Trondheim, Norway; 4 Department of Clinical and Molecular Medicine, Norwegian University of Science and Technology, Trondheim, Norway

**Keywords:** Brain metastases, Hybrid PET/MRI, Amino acid tracers, ^18^F-FACBC, Stereotactic, Radiotherapy, Recurrence

## Abstract

**Background:**

Patients with metastatic cancer to the brain have a poor prognosis. In clinical practice, MRI is used to delineate, diagnose and plan treatment of brain metastases. However, MRI alone is limited in detecting micro-metastases, delineating lesions and discriminating progression from pseudo-progression. Combined PET/MRI utilises superior soft tissue images from MRI and metabolic data from PET to evaluate tumour structure and function. The amino acid PET tracer ^18^F-FACBC has shown promising results in discriminating high- and low-grade gliomas, but there are currently no reports on its use on brain metastases. This is the first study to evaluate the use of ^18^F-FACBC on brain metastases.

**Case presentation:**

A middle-aged female patient with brain metastases was evaluated using hybrid PET/MRI with ^18^F-FACBC before and after stereotactic radiotherapy, and at suspicion of recurrence. Static/dynamic PET and contrast-enhanced T1 MRI data were acquired and analysed. This case report includes the analysis of four ^18^F-FACBC PET/MRI examinations, investigating their utility in evaluating functional and structural metastasis properties.

**Conclusion:**

Analysis showed high tumour-to-background ratios in brain metastases compared to other amino acid PET tracers, including high uptake in a very small cerebellar metastasis, suggesting that ^18^F-FACBC PET can provide early detection of otherwise overlooked metastases. Further studies to determine a threshold for ^18^F-FACBC brain tumour boundaries and explore its utility in clinical practice should be performed.

## Background

In the field of neuro-oncology, advancing the descriptive breadth and precision of imaging techniques is of paramount importance for describing tumour growth and structure. This information is invaluable in determining a treatment plan, evaluating the efficacy of treatment and detecting tumour recurrence.

Estimations show that 10–40% of patients with extracranial malignant tumours develop brain metastases (Helseth et al. 2003). The average survival time for these patients is less than 6 months (Stelzer 2013; Liu et al. 2019). Furthermore, the incidence of brain metastases is increasing. This is likely a consequence of the prolonged survival, provided by improved systemic therapies of primary tumours and the increased use of imaging techniques such as magnetic resonance imaging (MRI) (Liu et al. 2019; Nayak et al. 2012).

Structural MRI is used as a standard procedure to delineate, diagnose and plan treatment for brain metastases. MRI provides excellent soft tissue contrast, does not involve exposure to radiation and is an especially versatile imaging technique. However, MRI has some limitations, such as micro-metastasis detection, lesion boundary delineation and discrimination between progression and pseudo-progression after treatment (Bogsrud et al. 2019). Dynamic and static data acquired from amino acid (AA) positron emission tomography (PET) could provide the opportunity for a more detailed analysis of brain tumour extent and malignancy (Karlberg et al. 2017; Karlberg et al. 2019). Additionally, AA PET has been shown to detect malignant glioma progression as well as treatment efficacy earlier than MRI (Galldiks et al. 2013; Unterrainer et al. 2016). However, treatment planning using either PET or MRI can underestimate brain metastasis margins (Gempt et al. 2015). These MRI limitations might be reduced or even resolved with the utilisation of PET/MRI (Bogsrud et al. 2019; Galldiks et al. 2017). The combination of PET and MRI is a promising, non-invasive and relatively novel method. It can improve the detection of brain tumours, in addition to advancing precision and navigation during surgical and radiotherapy treatment (Karlberg et al. 2017; Karlberg et al. 2019). Hence, combined PET/MRI data can provide physicians with a better overview in the diagnosis of gliomas and brain metastases and influence clinical decision-making.

Some AA tracers 3,4dihydroxy-6-[^18^F]-fluoro-L-phenylalanine (^18^F-FDOPA), [^11^C]-methyl-methionine (^11^C-MET) and [^18^F]-fluoro-ethyltyrosin (^18^F-FET) are now recommended by international guidelines as a complement to MRI in the evaluation of gliomas (Albert et al. 2016). In contrast to ^18^F-2-fluoro-2-deoxy-D-glucose (^18^F-FDG), AA PET tracers show low uptake in healthy brain tissue and increased uptake in tumour cells, resulting in high tumour-to-background ratios (TBRs)—possibly due to an increased expression of AA transporters in tumour cells (Galldiks et al. 2017; Papin-Michault et al. 2016). A few studies also show promising results using ^11^C-MET and ^18^F-FET in patients with brain metastases, by reflecting biologic tumour behaviour, delineating tumour margins and differentiating progression from pseudo-progression after treatment (Gempt et al. 2015; Grosu et al. 2011; Unterrainer et al. 2017; Akhoundova et al. 2020).

Anti-1-amino-3-[18F]fluorocyclobutane-1-carboxylic acid (^18^F-FACBC) is another AA PET tracer with even higher TBRs than other AA PET tracers, which could aid the differentiation between high-grade and low-grade gliomas (Bogsrud et al. 2019; Karlberg et al. 2017; Karlberg et al. 2019). Furthermore, ^18^F-FACBC can reveal satellite tumours not detected with MRI in high-grade gliomas (Bogsrud et al. 2019). However, no studies have been performed using ^18^F-FACBC in patients with brain metastases.

The role of ^18^F-FACBC PET in the field of neuro-oncology remains to be thoroughly explored. This case report includes the analysis of four sequential ^18^F-FACBC PET/MRI examinations and investigates their utility in evaluating functional and structural brain metastasis properties.

## Case presentation

A middle-aged woman was treated for locally advanced breast cancer (invasive ductal carcinoma, ER+, PgR−, HER2) in 2012. Treatment included neoadjuvant chemotherapy, surgery, adjuvant radiation and hormonal therapy—with curative intent. After 2 more years, she was diagnosed with extracranial metastases and a solitary brain metastasis in the left frontal lobe. The brain metastasis was removed with gross total resection, followed by 18 weekly cycles of chemotherapy. Almost 3 years later, she presented with an 11-mm metastasis in the lateral precentral gyrus of the right brain hemisphere and a 30-mm metastasis in the cerebellum. The cerebellar metastasis was removed with gross total resection and the cerebral metastasis was treated with stereotactic radiotherapy (9 Gy × 3). As part of this study, four ^18^F-FACBC PET/MRI scans were conducted approximately 1 month apart on a simultaneous PET/MRI scanner (Siemens Biograph mMR, Siemens Healthcare, Erlangen, Germany). Scans 1 and 2 were conducted prior to and 1 month after stereotactic treatment of the cerebral metastasis, respectively. Scan 3 was conducted upon suspicion of a recurrence and showed a 9-mm recurrent cerebellar metastasis that was treated with stereotactic radiotherapy (9 Gy × 3). Scan 4 was conducted approximately 1 month after stereotactic radiotherapy treatment of the cerebellar metastasis (Table 1).

**Table 1:** Relevant patient medical history and interventions prior to and after PET/MRI acquisitions

**Relevant medical history and interventions prior to PET/MRI acquisitions**		
**Date**	**Findings**	**Interventions**
2012	Advanced breast cancer	Neoadjuvant chemotherapy, surgery, adjuvant radiation and hormonal therapy
2016	Extracranial metastases and a solitary brain metastasis in the left frontal lobe	Brain metastasis removed with gross total resection, followed by chemotherapy
2020	11-mm cerebral metastasis	Cerebellar metastasis removed with gross total resection
	30-mm metastasis in the cerebellum	Included in PET/MRI study
**PET/MRI scans included in the study**		
**Date**	**PET/MRI scan nr**	**Interventions**
Jan-20	Scan 1—prior to stereotactic radiotherapy of cerebral metastasis	Stereotactic radiotherapy of cerebral metastasis
Mar-20	Scan 2—follow-up 1 month after stereotactic radiotherapy of cerebral metastasis	
Apr-20	Scan 3—upon suspicion of recurrence of cerebellar metastasis	Stereotactic radiotherapy of cerebellar metastasis
Jun-20	Scan 4—follow-up 1 month after stereotactic radiotherapy of cerebellar metastasis	
**Relevant medical history and interventions after the PET/MRI acquisitions**		
**Date**	**MRI**	**Interventions**
Jul-20	Follow up MRI	Gross total resection of cerebral tumour

MRI sequences acquired included contrast-enhanced 3D T1, 3D FLAIR and ultrashort echo time for attenuation correction. For each of the four scans, the patient was required to fast for a minimum of 4 h prior to the injection of 189.7±8.0 MBq (3 MBq/kg) ^18^F-FACBC. Intravenous tracer injection occurred at *t* = 0 of the 35-min scan. PET tracer uptake was recorded in list/dynamic mode. PET image reconstruction was performed on the mMR with iterative reconstruction (3D OSEM algorithm, 3 iterations, 21 subsets, 344 matrix, 4-mm Gaussian filter) with point spread function, decay and scatter correction. MR-based attenuation correction was performed with a deep learning-based method (DeepUTE) using the ultrashort echo time MR sequence as input for making modified MR-based AC maps (Ladefoged et al. 2020).

### Static PET analysis

Data analysis was conducted on both lesions using PMOD software (version 4.104, PMOD Technologies LLC, Zürich, Switzerland) and was identical for data acquired from the four scans. Standard uptake values (SUVs) were evaluated using the patient’s body weight (SUV_bw_) from static PET images. Spherical volumes of interest (VOIs) were placed to encompass the lesion to calculate tumour SUV_max_ and SUV_peak_. A 2-mL VOI was placed on the contralateral side of the brain to calculate background SUV (SUV_bkg_). Using Eq. 1, peak and max tumour-to-background ratios (TBRs) were calculated for static PET images. TBR_peak_ was calculated for the cerebral tumour. Due to its small volume, only TBR_max_ was calculated for the cerebellar tumour.

1 $$ {\displaystyle \begin{array}{l}\left({TBR}_x={SUV}_{x\;(tumour)}/{SUV}_{mean\;(background)}\right)\\ {}x=\mathit{\max}\  or\ peak\end{array}} $$

**Fig. 1:**
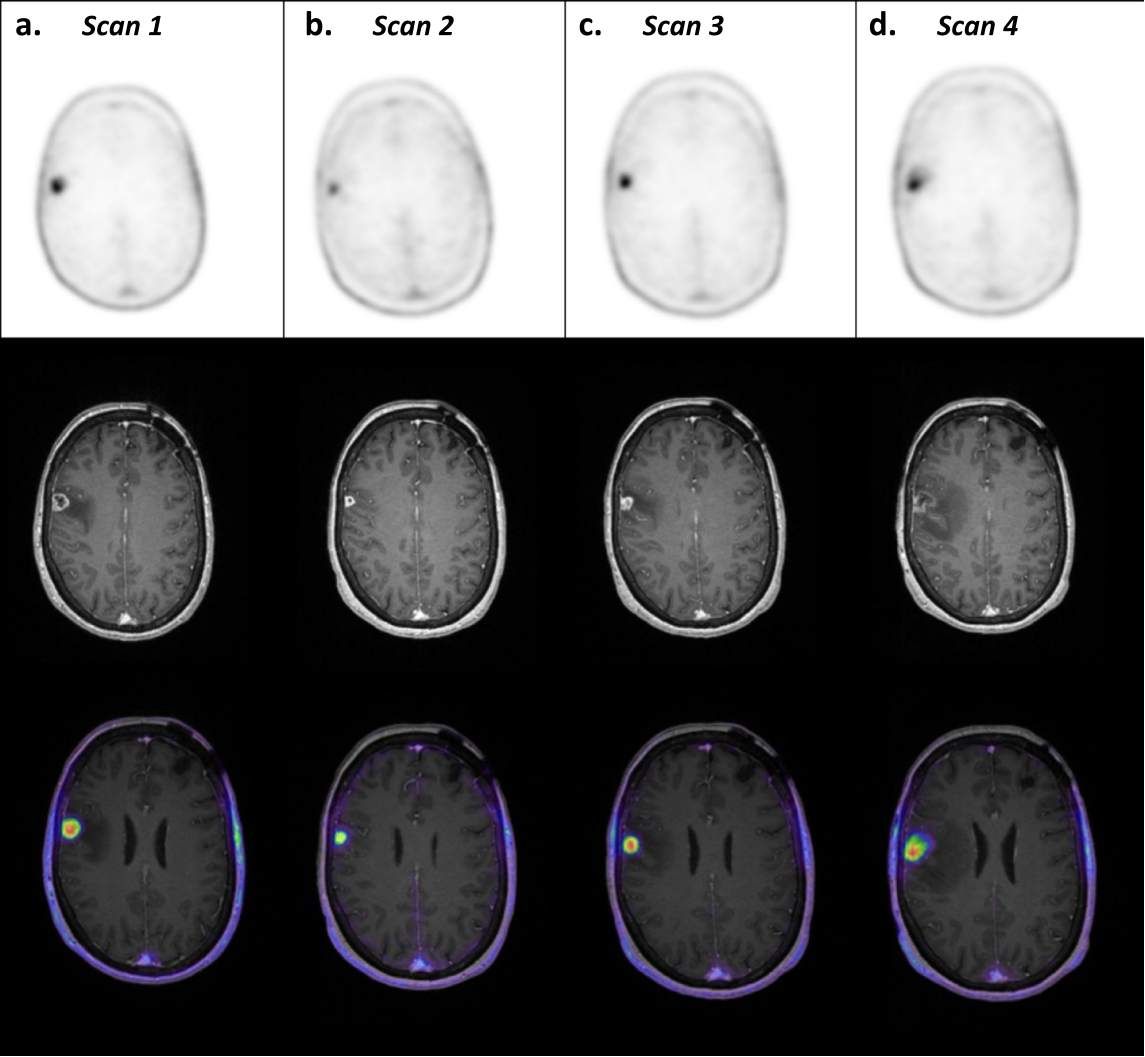
Static PET, T1 MRI_CE_ and fused PET/MR images of the cerebral tumour from scans 1–4. Axial view of the patient’s metastasis in the lateral precentral gyrus of the right hemisphere acquired using hybrid PET/MR imaging at four time points: scan 1—before stereotactic radiation, scan 2—1 month after the end of stereotactic radiation, scan 3—on suspicion of recurrence 1 month after scan 2, and scan 4—1 month after scan 3. Static PET (20–35 min), T1 MRI_CE_ and fused PET/MR images are shown from the top to bottom rows, respectively. Columns **a**–**d** show scans 1–4. The SUV scale for static PET images was 0–3.3, and the SUV scale of fused PET/MR images was 0.45–3.3

**Fig. 2:**
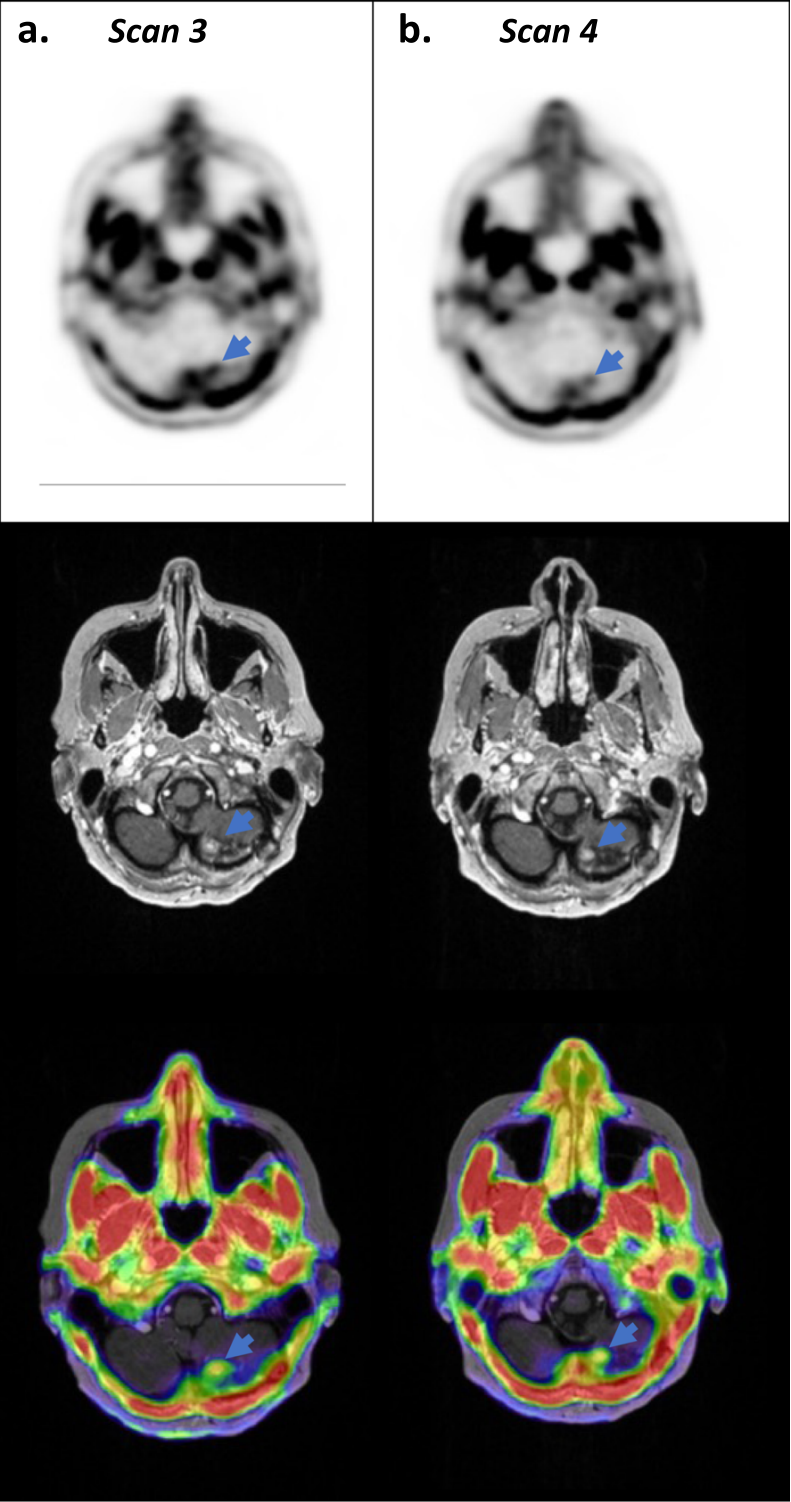
Static PET, T1 MRI_CE_ and fused PET/MR images of the cerebellar tumour in scans 3 and 4. Axial view of the patient’s left hemisphere caudomedial cerebellar metastasis (arrow) acquired using hybrid PET/MR imaging at time points 3 and 4. Static PET (20–35 min), T1 MRI_CE_ and fused PET/MR images are shown from the top to bottom rows, respectively. Columns **a** and **b** show scans 3 and 4. The SUV scale for static cerebellar PET images was 0–2, and the SUV scale of fused PET/MR images was 0.45–2

All four PET scans revealed a metastasis in the caudal part of the precentral gyrus of the right brain hemisphere (Fig. 1), while the latter two scans revealed a metastasis caudomedially in the left cerebellar hemisphere (Fig. 2). All scans exhibited low background ^18^F-FACBC uptake and high tumour ^18^F-FACBC uptake, with significantly higher uptake in the cerebral tumour compared to the cerebellar tumour (Tables 2 and 3). Cerebral tumour TBR_peak_ decreased between the first two scans following stereotactic radiotherapy and increased back to the initial values for scans 3 and 4. TBR_peak_ was the largest in scan 1. Cerebellar tumour TBR_max_ decreased between scans 3 and 4 (Table 3).

**Table 2:** Summary of static PET and volumetric PET/MRI cerebral tumour data for all four scans

	SUV_**bkg**_ [g/ml{SUVbw}]	SUV_**max**_ [g/ml{SUVbw}]	SUV_**peak**_ [g/ml{SUVbw}]	TBR_**peak**_	PET volume [ccm]	MRI volume [ccm]	PET/MRI intersect volume [ccm]
*Scan 1*	0.37	3.18	2.08	5.62	4.29	1.68	1.66
*Scan 2*	0.48	2.10	1.23	2.57	1.29	0.47	0.43
*Scan 3*	0.38	3.28	1.79	4.73	2.75	0.74	0.73
*Scan 4*	0.40	2.99	2.06	5.09	7.91	3.00	2.98

**Table 3:** Summary of static PET and volumetric PET/MRI cerebellar tumour data for scans 3 and 4

	SUV_**bkg**_ [g/ml{SUVbw}]	SUV_**max**_ [g/ml{SUVbw}]	TBR_**max**_	PET volume [ccm]	MRI volume [ccm]	PET/MRI intersect volume [ccm]
*Scan 3*	0.44	1.78	4.04	0.68	0.31	0.25
*Scan 4*	0.50	1.36	2.74	0.17	0.19	0.12

Throughout scans 2–4, it was difficult to distinguish viable tumour tissue from tissue reactions to radiotherapy in both metastases with clinical MRI. An MRI 1 month after scan 4 showed an increase in lesion volume, the patient was treated with a gross total resection of the cerebral tumour and histological findings confirmed tumour progression.

### Dynamic PET analysis

**Fig. 3:**
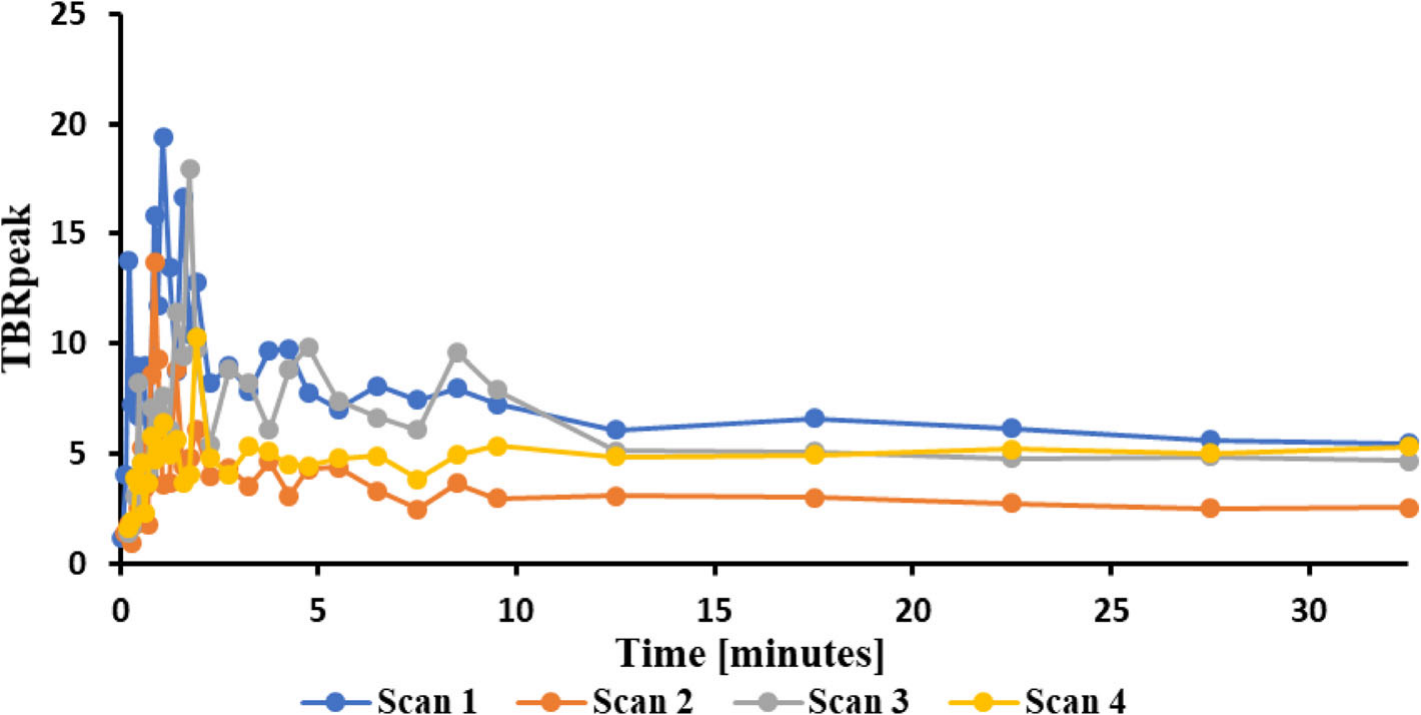
Graph showing cerebral tumour TBR_peak_ as a function of time for all four scans. TBR_peak_ values for each of the four scans peak within the first 2 min, followed by a decrease and a steady-state after 10 min

The tumour VOIs generated for static PET analysis were also used to calculate TBR_peak_ for dynamic data. Due to the smaller size of the cerebellar lesion, dynamic PET analysis was only conducted on the cerebral lesion. Dynamic PET data for all four scans showed that TBR_peak_ in the cerebral tumour peaked within the first 2 min after injection, at 65, 52.5, 105, and 115 s, respectively (Fig. 3). TBR_peak_ reached a steady-state after 10 min and was sustained for the remainder of the 35-min scan.

### Tumour volumes

**Fig. 4:**
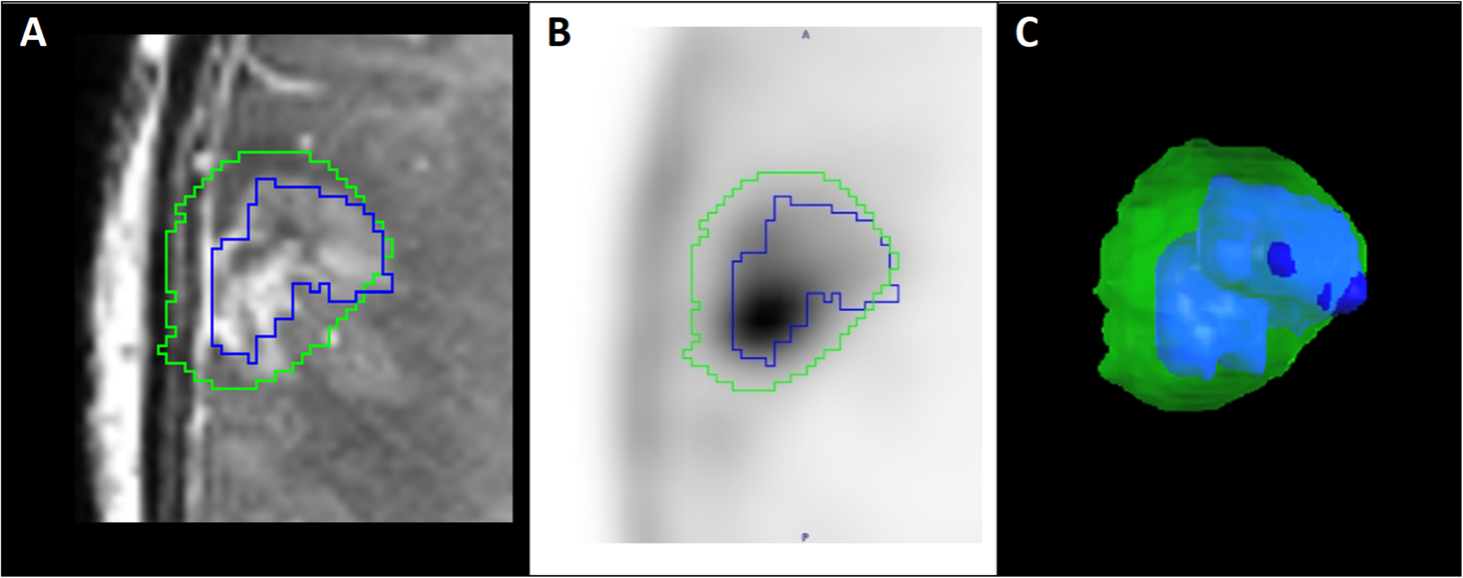
PET and MRI VOIs of cerebral tumour from scan 4. An example of the relationship between PET (green) and MRI (blue) tumour volumes that occurred in all four scans. **a** Zoomed in axial view of T1 MRI_CE_ image and **b** PET image from scan 4. **c** 3D representation showing PET VOI encompassing MRI_CE_ VOI from scan 4

For the PET and MRI tumour volume calculations, static PET (20–35 min) images were registered to T1 MRI_CE_ images in PMOD in order to get a perfect alignment between the two image modalities. PET tumour volumes were calculated by generating three-dimensional iso-contours inside a spherical VOI placed to encircle the entire tumour PET tracer uptake region. Iso-contour boundaries were defined as the region with PET tracer uptake > 2 × SUV_bkg_, and regions of high non-tumour PET tracer uptake were manually erased. T1 MRI_CE_ tumour volumes were determined by manually tracing tumour boundaries for each axial slice. Intersecting PET and MRI tumour volumes were calculated in PMOD using PET and MRI tumour volume VOIs. For all time points, the PET volumes were larger than, and enclosed, T1 MRI_CE_ volumes (Tables 2 and 3) for the cerebral tumour (see example, Fig. 4). Both PET- and MRI-based cerebral tumour volumes decreased between scans 1 and 2, followed by a slight increase in scan 3 and then an increase to almost 2× initial values in scan 4 (Table 2). Cerebral TBR_peak_ values correlated positively with PET and MRI volume changes for the first two scans (Table 2) but returned to initial values in scans 3 and 4. Relative PET and MRI volume changes were near identical and followed the same trend throughout the four scans.

Both PET- and MRI-based cerebellar tumour volumes decreased between scans 3 and 4 (Table 3), and this coincided with a decrease in tumour TBR_max_ following stereotactic radiotherapy. However, the PET volume showed a relatively larger decrease than the MRI volume, resulting in a slightly smaller PET than MRI volume in scan 4.

## Discussion

This is the first study aimed to evaluate ^18^F-FACBC performance of brain metastases using four consecutive PET/MRI examinations. The results of this study show that ^18^F-FACBC could provide useful information with regard to tumour detection. In particular, there was low ^18^F-FACBC tracer uptake (SUV_bkg_) in healthy brain tissue as well as high tracer uptake (SUV_peak_ and SUV_max_) (Tables 2 and 3) in both cerebral and cerebellar tumour VOIs, consistent with previous studies on gliomas (Bogsrud et al. 2019; Karlberg et al. 2017; Karlberg et al. 2019; Tsuyuguchi et al. 2017; Michaud et al. 2020). We reported an increase in ^18^F-FACBC uptake in healthy brain tissue (SUV_bkg_) after radiation therapy in scan 2 (Table 2), but regardless of this increase, the distinctively low ^18^F-FACBC tracer uptake in healthy tissue (SUV_bkg_) played a large role in the high tumour contrast (TBR_max_ and TBR_peak_) in this patient. Previous studies using AA tracers ^11^C-MET and ^18^F-FET on brain metastases reported higher SUV_bkg_ and on average lower TBR_max_ values than ^18^F-FACBC in the current study (Gempt et al. 2015; Grosu et al. 2011; Ceccon et al. 2017), probably caused by differences in the transport system used by the various AA tracers. ^11^C-MET and ^18^F-FET rely primarily on the system L amino acid transporter (LAT1) for accumulation in cancerous cells (Sun et al. 2018; Heiss et al. 1999; Habermeier et al. 2015; Ono et al. 2013), while ^18^F-FACBC utilises both LAT1 and the alanine-serine-cysteine transporter 2 (ASCT2) (Ono et al. 2013; Okudaira et al. 2015). Since ^18^F-FACBC has a higher affinity for ASCT2 than LAT1, and since ASCT2 is not expressed on the luminal side of the blood vessels, this could potentially lead to a lower transport of ^18^F-FACBC compared to ^18^F-FET and ^11^C-MET across the intact BBB in healthy tissue. In line with these results, Michaud et al. (Michaud et al. 2020) have indeed demonstrated that the transport across the intact BBB in healthy tissue in patients with recurrent gliomas was around 6 times higher for MET compared to ^18^F-FACBC. In tumour tissue with a broken BBB, the transport will be increased for all the AA-tracers. But since ^18^F-FACBC mainly relies on ASCT2 transport, which is expressed on the abluminal side of the blood vessels, this could further increase the uptake for ^18^F-FACBC compared to ^18^F-FET and ^11^C-MET in tumour tissue with a broken BBB, further increasing the TBR seen for ^18^F-FACBC. It has been shown that ASCT2 is likely more active in early stages of tumour development, whereas LAT1 predominates with increasing acidity from the cell density that increases with tumour progression (Oka et al. 2012). This could indicate that ^18^F-FACBC is a better AA PET tracer in detecting early stages of tumour development, while ^11^C-MET and ^18^F-FET uptake correlates more with advanced tumour progression and recurrence than ^18^F-FACBC. Further studies should be conducted to elucidate ^18^F-FACBC pharmacokinetics and how it is affected by pathological cellular changes, as well as evaluate the promising contrast provided by ^18^F-FACBC in brain metastases.

Notably, the cerebellar tumour was detected by ^18^F-FACBC PET in scans 3 and 4, despite having a volume of less than 0.2 ccm in scan 4 (Table 3). This is particularly noteworthy as it supports a previous study which reported that the high TBR provided by ^18^F-FACBC PET allowed the detection of small satellite tumours that were not registered by MRI (Bogsrud et al. 2019). This is a promising result with regard to early detection of brain metastases that might otherwise be overlooked.

Dynamic evaluation of the cerebral tumour TBR_peak_ showed a time-to-peak (TTP) of less than 2 min for all scans, followed by a decrease reaching a steady-state within 10 min which persisted for the remainder of the examination. This steady-state provided an ideal time frame for static PET analysis, justifying the 20–35-min time frame used in this study, and is comparable to the 10–20-min time frame recommended by guidelines for static AA PET analysis using ^11^C-MET and ^18^F-FET (Law et al. 2019). These findings are consistent with ^18^F-FACBC uptake in high-grade gliomas reported by Karlberg et al. and Kondo et al. which described a steady-state following a TTP of 43 s and < 10 min, respectively (Karlberg et al. 2019; Kondo et al. 2016). The similarity between brain metastasis and glioma AA PET uptake patterns should be further explored to better understand tumour physiology and AA PET tracer uptake.

Studies have suggested that dynamic ^18^F-FET uptake can be used for tumour grading, where a slow increase in the dynamic uptake in low-grade gliomas and a short TTP followed by a decrease in the high-grade gliomas has been demonstrated (Pöpperl et al. 2007). The dynamic ^18^F-FET PET analysis in patients with brain metastases has on the other hand demonstrated a broad distribution of TTP and time-activity curves throughout metastases of different primary tumours (Unterrainer et al. 2017). In this study investigating brain metastases from breast cancer, TTP and time-activity curves using ^18^F-FACBC showed the same pattern across all four scans regardless of radiotherapy, but no conclusions with regard to its diagnostic value can be reached based on the paucity of data of ^18^F-FACBC uptake analysis in metastases.

When investigating pathology in the brain, inadequate transport across the intact BBB can lead to limited diagnostic performance and the production of erroneous results, invalidating the usefulness of the tracer as a supplement to contrast-enhanced MRI. MR contrast agents do not cross the intact BBB, and as already noted, the transport across the intact BBB is shown to be extremely low for FACBC (Michaud et al. 2020). Nevertheless, ^18^F-FACBC uptake has been observed in some progressive and recurrent gliomas that were not defined with contrast-enhanced MRI (Bogsrud et al. 2019; Michaud et al. 2020), and it has also been shown that ^18^F-FET PET tumour margins differed significantly from those calculated from MRI in patients with brain metastases (Gempt et al. 2015). This suggests that CE and AA tracer uptake varies when it comes to transport across the intact and broken BBB and that combined PET/MRI data may help describe more exact brain metastasis margins. In this study, tumour boundaries were defined by regions with PET tracer uptake >2 × SUV_bkg_. When using this threshold, PET volumes were consistently larger than and enclosed the vast majority of T1 MRI_CE_ volumes. However, compared to ^18^F-FET-examinations, where the thresholds for defining FET-based volumes have been verified with histological analysis (Pauleit et al. 2005; Law et al. 2019), there are no established guidelines for which threshold that should be used for ^18^F-FACBC PET volumetric brain tumour analysis. Because of this, the usefulness of ^18^F-FACBC PET/MRI in the volumetric tumour analysis of brain metastases cannot yet be determined.

In this study, the small cerebellar metastasis was discovered simultaneously in scan 3 with PET and MRI. Due to a lack of studies, it was uncertain whether ^18^F-FACBC uptake would correspond to radiotherapy-induced pseudo-progression or actual tumour progression—as has been shown with ^11^C-MET and ^18^F-FET (Grosu et al. 2011; Akhoundova et al. 2020). In this case, however, the latter was found to be true; gross total resection and histology of the cerebral tumour confirmed tumour progression—a distinction not possible to make with MRI alone.

There are clear limitations to this study, such as the limited sample size of only one patient and its low reproducibility due to a lower availability of PET/MRI than PET/CT. There are also limitations in the MR-based method used for attenuation correction purpose in postoperative patients with craniotomies and metal implants, and in children, since the method for the updated software is only evaluated in adults (Ladefoged et al. 2020). However, the patient in the current study did not have any implants in the brain that could interfere with the MR-based attenuation correction method, and this issue should therefore not affect the results. Also, there are limitations for calculating ^18^F-FACBC-based tumour volumes, since no established thresholds exist for this tracer in brain tumours yet. More studies should be performed to correlate ^18^F-FACBC uptake with histological findings to determine appropriate thresholds, and to further investigate whether ^18^F-FACBC can help delineate tumour spread more accurately compared to MRI only.

## Conclusion

This study describes a potentially promising use for ^18^F-FACBC PET in brain metastases. ^18^F-FACBC provided high TBR_peak_ values in brain metastases, mostly due to a consistently low tracer uptake in healthy tissue (SUV_bkg_). This shows that ^18^F-FACBC may be used as a tool for visualising brain metastases effectively. There was even high ^18^F-FACBC uptake in the small cerebellar metastasis, which suggests that ^18^F-FACBC could play an important role in detecting brain metastases that might otherwise be overlooked. However, more studies are needed to correlate ^18^F-FACBC PET uptake with histological findings to determine appropriate thresholds for ^18^F-FACBC-based brain tumour volume definition.

## Data Availability

The datasets used and/or analysed during the current study are available from the corresponding author on reasonable request.

## Abbreviations

^18^F-FACBC: Anti-1-amino-3-[^18^F]-fluorocyclobutane-1-carboxylic acid
PET: Positron emission tomography
MRI: Magnetic resonance imaging
AA: Amino acid
^18^F-FDOPA: 3,4Dihydroxy-6-[^18^F]-fluoro-L-phenylalanine
^11^C-MET: [^11^C]-methyl-methionine
^18^F-FET: [^18^F]-fluoro-ethyltyrosin
^18^F-FDG: ^18^F-2-fluoro-2-deoxy-D-glucose
TBR: Tumour-to-background ratio
FLAIR: Fluid-attenuated inversion recovery
SUV: Standard uptake value
VOI: Volume of interest
LAT1: System L amino acid transporter 1
ASCT2: Alanine-serine-cysteine transporter 2
